# The role of human Metapneumovirus genetic diversity and nasopharyngeal viral load on symptom severity in adults

**DOI:** 10.1186/s12985-018-1005-8

**Published:** 2018-05-23

**Authors:** Xiang Yong Oong, Jack Bee Chook, Kim Tien Ng, Wei Zhen Chow, Kok Gan Chan, Nik Sherina Hanafi, Yong Kek Pang, Yoke Fun Chan, Adeeba Kamarulzaman, Kok Keng Tee

**Affiliations:** 1 Department of Medicine, Faculty of Medicine, University of Malaya, 50603 Kuala Lumpur, Malaysia; 2 School of Healthcare and Medical Sciences, Sunway University, 47500 Kuala Lumpur, Selangor Malaysia; 3 Division of Genetics and Molecular Biology, Institute of Biological Sciences, Faculty of Science, University of Malaya, 50603 Kuala Lumpur, Malaysia; 4 Department of Primary Care Medicine, Faculty of Medicine, University of Malaya, 50603 Kuala Lumpur, Malaysia; 5 Department of Medical Microbiology, Faculty of Medicine, University of Malaya, 50603 Kuala Lumpur, Malaysia

**Keywords:** Human metapneumovirus (HMPV), Genetic diversity, Viral load, Symptom severity, Acute respiratory tract infection

## Abstract

**Background:**

Human metapneumovirus (HMPV) is established as one of the causative agents of respiratory tract infections. To date, there are limited reports that describe the effect of HMPV genotypes and/or viral load on disease pathogenesis in adults. This study aims to determine the role of HMPV genetic diversity and nasopharyngeal viral load on symptom severity in outpatient adults with acute respiratory tract infections.

**Methods:**

Severity of common cold symptoms of patients from a teaching hospital was assessed by a four-category scale and summed to obtain the total symptom severity score (TSSS). Association between the fusion and glycoprotein genes diversity, viral load (quantified using an improved RT-qPCR assay), and symptom severity were analyzed using bivariate and linear regression analyses.

**Results:**

Among 81/3706 HMPV-positive patients, there were no significant differences in terms of demographics, number of days elapsed between symptom onset and clinic visit, respiratory symptoms manifestation and severity between different HMPV genotypes/sub-lineages. Surprisingly, elderly patients (≥65 years old) had lower severity of symptoms (indicated by TSSS) than young and middle age adults (*p* = 0.008). Nasopharyngeal viral load did not correlate with nor predict symptom severity of HMPV infection. Interestingly, at 3–5 days after symptom onset, genotype A-infected patients had higher viral load compared to genotype B (4.4 vs. 3.3 log_10_ RNA copies/μl) (*p* = 0.003).

**Conclusions:**

Overall, HMPV genetic diversity and viral load did not impact symptom severity in adults with acute respiratory tract infections. Differences in viral load dynamics over time between genotypes may have important implications on viral transmission.

**Electronic supplementary material:**

The online version of this article (10.1186/s12985-018-1005-8) contains supplementary material, which is available to authorized users.

## Background

Human metapneumovirus (HMPV) is a negative-stranded RNA virus classified in the *Pneumoviridae* family [1]. HMPV infections are commonly associated with mild respiratory symptoms, but severe cough, bronchiolitis and pneumonia have also been reported, sometimes accompanied by high fever, myalgia and vomiting [2]. Several risk factors associated with more severe disease due to HMPV infection in adults have been identified, which include patients with pulmonary disease or congestive heart disease, healthy elderly patients with age over 65 years old, long term stay in hospital care facilities and immunocompromised patients [3].

Previous reports had attempted to associate the genetic diversity of HMPV, which are classified as genotypes A and B (with further classification into sub-lineages - A1, A2a, A2b, B1 and B2) [4] with disease severity [5–7]. For instances, it was found that HMPV genotype A infection in children caused more severe illnesses (e.g. higher risk of pneumonia and oxygen saturation < 90%, need for hospitalization, and longer stay of intensive care unit) compared with genotype B infection [7]. In contrast, some studies reported that genotype B infection was one of the risk factor for severe disease [5] with more pathological signs on chest X-ray compared with genotype A infection [6]. Furthermore, some associations remain debatable as several studies found no direct correlation between HMPV genotypes and severity of illness [8–10]. On the other hand, apart from viral genetic diversity, HMPV viral load was recognized as a risk factor associated with more severe disease outcome leading to hospitalization [11, 12].

Although HMPV genetic diversity continues to be described and linked with disease severity in hospitalized children [6], pediatrics [13], elderly adults [10] and immunocompromised patients [3], reports that address this association have been limited in the adult outpatient settings. A recent study which showed that HMPV can also cause respiratory outbreaks in adults [14] highlights the fact that adults may play a role in the transmission and evolutionary dynamics of the virus, and more severe disease could occur in adults during an outbreak.

Hence, in this study, we sought to investigate the possible linkage of genetic diversity on symptom severity in adult outpatients with HMPV infection presenting acute upper respiratory tract infections (URTI) [15]. Using an improved molecular assay for viral load quantification, we also assessed the correlation of HMPV viral load in nasopharyngeal specimens on symptom severity.

## Methods

### Sample collection, symptom severity assessment, and HMPV genotyping

A total of 3706 consenting adult outpatients who presented with symptoms of acute URTI for not more than two weeks were recruited at the Primary Care Clinic of University of Malaya Medical Centre (UMMC) in Kuala Lumpur, Malaysia between February 2012 and May 2014. During enrollment, participants were interviewed to determine their demographics (age, gender and ethnicity), estimated number of days elapsed between symptom onset and clinic visit or enrollment date, and the presence and severity of common cold symptoms [16, 17]. The common cold symptoms assessed were sneezing, nasal discharge, nasal congestion, cough, sore throat, hoarseness of voice, headache and muscle ache. The severity of each symptom was then rated by a standardized four-category ordinal scale previously reported [16–19]: none (0), mild (1), moderate (2) and severe (3). The symptom ratings were summed to create a total symptom severity score (TSSS) for each participant with a maximum points of 24 from eight symptoms [20], in which greater symptom severity was indicated by a higher score.

Nasopharyngeal swabs were collected from the patients and transferred to the laboratory in universal transport media (Copan Diagnostics, California, USA). Total nucleic acid purification was performed using the NucliSENS easyMAG automated nucleic acid extraction system (bioMérieux, Marcy I’Etoile, France) according to manufacturer’s protocol [21]. The xTAG Respiratory Virus Panel (RVP) FAST multiplex RT-PCR assay (Luminex Molecular, Toronto, Canada) and Luminex’s proprietary Universal Tag sorting system on Luminex 200 IS platform (Luminex Corp., Austin, Texas, USA) were used to detect HMPV in the samples [22]. The genotype of HMPV-positive samples was first determined by performing amplification and sequencing of the fusion (*F*) and attachment (*G*) genes as previously described [15]. This was followed by phylogenetic tree reconstruction using the maximum-likelihood (ML) method which was heuristically inferred using subtree pruning and regrafting and nearest neighbor interchange algorithms with a general time-reversible (GTR) nucleotide substitution model, a proportion of invariant sites (+I) and four categories of gamma rate heterogeneity (+Γ_4_), which were implemented in PAUP version 4.0 [23]. Kimura’s two-parameter model with a reliability of branching order analyzed by bootstrap replicates of 1000 was used.

### HMPV viral load quantification

For improved quantification of HMPV RNA in the nasopharyngeal specimens, a comprehensive and updated list of reference genomes was used to design a quantitative one-step reverse transcriptase-quantitative polymerase chain reaction (RT-qPCR) assay. Newly designed primer pair and a fluorescent probe targeting the highly conserved *M2* gene [24] of HMPV were developed based on a set of global HMPV complete genomes representing all genotypes A1, A2a, A2b, B1 and B2 (*n* = 135) available in GenBank (retrieved on 31 January 2016). The sequences were codon-aligned using the web-based multiple sequence alignment program MAFFT [25] to look for conserved regions of the complete genome. Highly conserved forward primer, reverse primer and the probe with a coverage of 99.3, 100, and 99.3%, respectively, based on the alignment of the global reference sequence (Additional file 1), were designed using the Primer Express Software v2.0 (Applied Biosystems, California, USA). With reference to the nucleotide numbering of NC_004148 (HMPV reference strain), the forward primer (designated as 4730f), reverse primer (4919r) and probe (4796fp) spanned the genetic regions corresponding to nucleotide positions 4730–4754, 4919–4893 and 4796–4814 nt, respectively. The probe was labeled with the 6-fluorescein amidite (FAM) at the 5′ end, and the non-fluorescent quencher (NFQ) and minor groove binder (MGB) at the 3′-end. A synthetic single-stranded RNA oligonucleotide with a randomly generated sequence was designed and used as internal control (IC) to check for potential PCR inhibition. The unique IC sequence (5′-ACATCGTAAGGCTCCATGCAAATATGAAGATAGAATGCTTAGGACCATCAGCGAAACTCTACAATAATATCAGGCGCAGGCAGAGAAGTA-3′) showed < 10% similarity with any published sequence in the GenBank (data not shown). The IC was flanked by sequences similar to the primer binding site of the newly designed HMPV primer set, with a unique non-HMPV probe designed for the IC (VIC-5′-TTAGGACCATCAGCGAAAC-3′-NFQ-MGB). In a single reaction, 0.2 μl of reverse transcriptase (40 U), 10 μl of 2× One-Step SensiFAST Probe Lo-ROX mix (Bioline, London, UK), 0.8 μl of each primer (20 μM), 1 μl of each probe (10 μM), and 6.0 × 10^3^ RNA copies/μl of IC in a final volume of 20 μl. The optimized thermal cycling profile used was as follows: reverse transcription at 48 °C for 8 min, initial denaturation at 95 °C for 2 min, followed by 40 cycles of 97 °C for 2 s, and 60 °C for 20s. The thermal cycling period of the assay was short, in which viral load quantification can be accomplished within approximately 50 min in a single run. A synthetic DNA oligonucleotide containing the *M2* gene sequence was used to generate a 10-fold dilution series of standard concentrations, ranging from 2.0 × 10^1^ to 2.0 × 10^6^ RNA copies/μl. HMPV quantification was performed in the ABI ViiA7 Real-time PCR System (Applied Biosystems, California, USA).

The linear dynamic range of the HMPV qPCR assay was assessed using a 6-log_10_ dilution series of HMPV *M2* synthetic oligonucleotide. A standard curve plotted against quantification cycle (Cq) was built using the serial concentration. Linear regression analysis was performed to calculate the PCR efficiency and correlation coefficient based on the standard curve. In order to assess the intra- and inter-assay variability for HMPV viral load quantification, triplicate reactions were performed using a low (2.0 × 10^1^ RNA copies/μl) and moderate (2.0 × 10^3^ RNA copies/μl) standard to determine the mean, standard deviation (SD) and coefficient of variance (CV).

### Statistical analysis

Demographic (sex, age and ethnicity) and clinical (presence of the eight common cold symptoms and estimated number of days elapsed between symptom onset and enrollment date) characteristics of patients infected by different HMPV genotypes (A and B) and sub-lineages were first assessed using the bivariate analyses (Pearson’s chi-square for categorical variables, Independent Samples *t*-test and One-way ANOVA for continuous variables), similar to statistical techniques previously reported [12]. The overall severity of the symptoms was measured through the summation of eight individual symptom severity score (TSSS), which was modeled as a continuous variable. Association of symptom severity with virological factors (infection by different HMPV genotypes and sub-lineages, and viral load in log_10_ RNA copies/μl) and demographic factors was performed using bivariate analyses (Independent Samples t-Test, One-way ANOVA, and Pearson’s bivariate correlation) and linear regression. Lastly, comparisons of viral load between different periods of enrollment after symptom onset (taking into account the infection by different genotypes/sub-lineages and the differences in demographics and symptom severity profiles) were made using similar analyses mentioned above. A two-sided *p* value lower than 0.05 was considered statistically significant. In order to control false positives in multiple statistical tests such as One-way ANOVA and linear regression, the Bonferroni correction was used to lower the critical *p* value of significance (performed by dividing the critical value by the number of comparisons corresponding to the number of levels in a group) [26]. All analyses were performed using the Statistical Package for Social Sciences version 22.0 (SPSS Inc., New York, USA).

## Results

### Detection and genetic diversity of HMPV

**Fig. 1:**
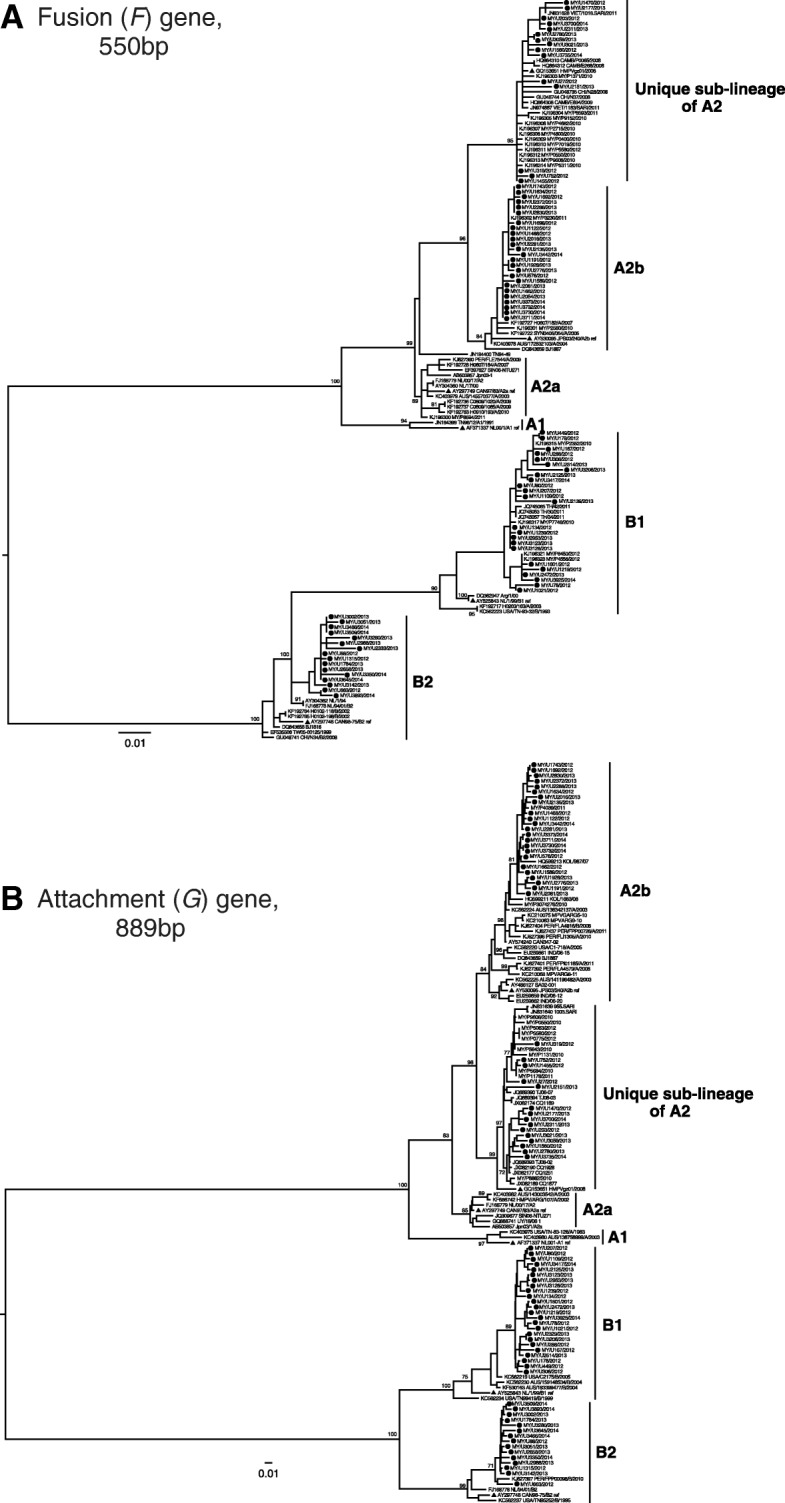
Phylogenetic analysis of **a**) 85 fusion (*F*) and **b**) 82 attachment (*G*) genetic sequences. Maximum-likelihood trees were reconstructed using PAUP version 4.0. The reliability of the branching nodes was assessed by bootstrap analysis of 1000 replicates. Bootstrap values of greater than 70% were indicated on the branch nodes. The generated sequences (dark circles) were named according to the country of isolation (Malaysia, MY), unique sample ID and year of sample collection. Published HMPV reference strains for each genotype/sub-lineage (blue triangles) included A1, NL00–1 (GenBank accession number: AF371337.1), A2a, CAN97–83 (AY297749.1), A2b, JPS03–240.1 (AY530095), B1, NL/1/99 (AY525843.1), and B2, CAN98–75 (AY297748.1). Other published sequences included those from Australia (AUS), Cambodia (CAMB), Canada (CA), India (IND), Japan (JP), Netherlands (NL), Peru (PER), Singapore (SIN), Thailand (TH), United States (USA), and Vietnam (VIET)

During the study period, a total of 81/3706 (2.2%) nasopharyngeal swab specimens collected were tested positive for HMPV. Among them, only 7/81 (8.6%) specimens were cases of coinfection with other viruses (adenovirus [*n* = 1], enterovirus/rhinovirus [*n* = 3], coronavirus 229E [n = 1], influenza A (H3) + enterovirus/rhinovirus [*n* = 1] and influenza B [*n* = 1]). Thus, as coinfection of HMPV viruses was not common in the adult population, we included patients with viral codetection in subsequent analyses and was not considered as another variable in this study. Phylogenetic analysis of the *F* and *G* genes showed that 40/81 (49.4%) of detected HMPV viruses belonged to genotype A whereas 41/81 (50.6%) belonged to genotype B (Fig. 1, Additional file 2). Within genotype A, 25/40 (62.5%) were classified as sub-lineage A2b, whereas 15/40 (37.5%) belonged to a recently described sub-lineage of A2 (designated as unique A2 sub-lineage) [15]. Within genotype B, 25/41 (61.0%) and 16/41 (39.0%) were classified as sub-lineages B1 and B2, respectively.

### Demographic and clinical association with HMPV genetic diversity

Eighty-one outpatients (29 males and 52 females) who were infected by HMPV had an age range of 19 to 86 years old, consisting of adults and elderly (Additional file 2). Among them, 65 patients were grouped as young and middle-age adults (< 65 years old), while 16 patients were grouped as elderly (≥65 years old) (Table 1). The majority of the patients were Malays (*n* = 38), followed by Indians (*n* = 21), Chinese (*n* = 19), and other ethnic groups (*n* = 3). When bivariate analyses were performed, no significant association was observed between HMPV genotypes (A and B) or sub-lineages (A2b, unique A2 sub-lineage, B1, and B2) with the patients’ demographics (sex, age, and ethnicity).

**Table 1:** Demographic and clinical characteristics of HMPV infection caused by different genotypes and sub-lineages

	HMPV Genotype			HMPV Sub-lineage				
Characteristics	A, *n* = 40	B, *n* = 41	*p*	A2b, *n* = 25	Unique A2 sub-lineage, *n* = 15	B1, *n* = 25	B2, *n* = 16	*p*
Sex								
Male, *n* = 29	13	16	0.540^b^	8	5	11	5	0.784^b^
Female, *n* = 52	27	25		17	10	14	11	
Age								
< 65 years old, *n* = 65	32	33	0.956^b^	20	12	22	11	0.516^b^
≥ 65 years old, *n* = 16	8	8		5	3	3	5	
mean (±SD)	45.28 ± 18.26	46.15 ± 20.12	0.839^a^	44.36 ± 18.68	46.80 ± 18.07	44.56 ± 17.63	48.63 ± 23.91	0.892^c^
Ethnicity								
Chinese, *n* = 19	12	7	0.402^b^	6	6	2	5	0.144^b^
Malay, *n* = 38	18	20		15	3	14	6	
Indian, *n* = 21	8	13		3	5	9	4	
Others, *n* = 3	2	1		1	1	0	1	
Presence of Symptoms								
Sneezing, *n* = 56	29	27	0.517^b^	20	9	18	9	0.346^b^
Nasal congestion, *n* = 54	23	31	0.084^b^	14	9	21	10	0.166^b^
Nasal discharge, *n* = 58	31	27	0.245^b^	20	11	16	11	0.645^b^
Cough, *n* = 80	40	40	0.320^b^	25	15	24	16	0.519^b^
Sore throat, *n* = 59	28	31	0.570^b^	19	9	21	10	0.282^b^
Hoarseness of voice, *n* = 72	36	36	0.753^b^	23	13	23	13	0.680^b^
Muscle ache, *n* = 56	26	30	0.426^b^	17	9	18	12	0.812^b^
Headache, *n* = 57	27	30	0.576^b^	15	12	18	12	0.541^b^
Estimated no. of days elapsed between symptom onset and enrollment date								
≤ 1–2 days, *n* = 20	9	11	0.207^b^	5	4	7	4	0.600^b^
3–5 days, *n* = 36	15	21		11	4	12	9	
≥ 6 days, *n* = 25	16	9		9	7	6	3	
mean (±SD)	5.15 ± 3.63	4.12 ± 2.55	0.143^a^	4.56 ± 2.04	6.13 ± 5.28	4.20 ± 2.93	4.00 ± 1.80	0.211^c^

*n:* number of patients, *SD:* standard deviation

^a^*p*-value calculated by Independent Samples t-Test

^b^p-value calculated by Pearson’s Chi-square test

^c^p-value calculated by One-way ANOVA

*p* = level of significance (2 tailed) at the 0.05 level

The presence of common cold symptoms assessed in this study (sneezing, nasal discharge, nasal congestion, cough, sore throat, hoarseness of voice, muscle ache and headache) was self-reported by the patients. The majority of the patients experienced cough (*n* = 80/81, 98.8%) followed by hoarseness of voice (*n* = 72/81, 88.9%), while muscle ache and sneezing (*n* = 56/81, 69.1%) were the least experienced by patients, respectively (Table 1). No significant differences were noted in the manifestation of all symptoms between different genotypes or between different sub-lineages. Though, there were noticeably more HMPV genotype B patients (*n* = 31/41, 75.6%) that experienced nasal congestion compared to genotype A patients (*n* = 23/40, 57.5%) (*p* = 0.084). The estimated number of days elapsed between symptom onset and enrollment date as reported by the patients ranged from 1 day to 2 weeks, where most patients were enrolled after a symptomatic period of 3–5 days (*n* = 36/81, 44.4%) (Table 1). While no significant association was found between different enrollment periods after symptom onset and HMPV genotypes or sub-lineages, it was observed that HMPV genotype A-infected patients enrolled slightly later after symptom onset (5.15 ± 3.63 days) compared to genotype B patients (4.12 ± 2.55 days) (*p* = 0.143).

### Symptom severity of HMPV-infected patients

A greater perceived symptom severity of HMPV-infected patients was indicated by a higher TSSS, which was calculated by the summation of eight individual symptom severity score as reported or assessed during enrollment [20]. Overall, the infected patients had a mean TSSS of 12.20 ± 4.58, with a range of score from 4 to 23 (maximum score is 24) (Additional file 2), indicating that patients may experience a range of mild to severe respiratory illnesses. When TSSS was compared between different HMPV genotypes and sub-lineages by bivariate and linear regression analyses, no significant differences were observed, even though genotype B patients reported higher TSSS (12.85 ± 4.67) compared to genotype A patients (11.53 ± 4.44), with sub-lineage B1 patients had the highest TSSS (13.56 ± 4.42) among the sub-lineages (Table 2). Also, no significant differences of mean TSSS were observed between different periods of enrollment after symptom onset and no correlation with TSSS was found when the days of enrollment after symptom onset was treated as a continuous variable. The mean TSSS between different sexes and between ethnic groups was not significantly different as well, even though male patients had a higher TSSS compared to female while the Indian ethnic group had the highest TSSS compared to other ethnic groups (Table 2).

**Table 2:** Assessment of demographical and virological predictors for symptom severity in HMPV-infected patients

Characteristics	TSSS (mean ± SD)	*p*	*β*	p*
HMPV genotype				
A, *n* = 40	11.53 ± 4.44	0.194^a^	1.329	0.194
B, *n* = 41	12.85 ± 4.67		*ref.*	
HMPV sub-lineage				
A2b, *n* = 25	11.16 ± 4.12	0.306^b^	*ref.*	
Unique A2 sub-lineage, *n* = 15	12.13 ± 5.03		0.973	0.515
B1, *n* = 25	13.56 ± 4.42		2.400	0.067
B2, *n* = 16	11.75 ± 4.99		0.590	0.687
Estimated no. of days elapsed between symptom onset and enrollment date	*r* = 0.106	0.348^c^	0.153	0.348
≤ 1–2 days, *n* = 20	10.95 ± 4.77	0.373^b^	− 1.578	0.221
3–5 days, *n* = 36	12.53 ± 4.35		*ref.*	
≥ 6 days, *n* = 25	12.72 ± 4.75		0.192	0.872
Sex				
Male, *n* = 29	12.41 ± 4.73	0.753^a^	− 0.337	0.753
Female, *n* = 52	12.08 ± 4.54		*ref.*	
Age	*r* = −0.335	**0.002** ^**c**^	− 0.080	**0.002**
< 65 years old, *n* = 65	12.86 ± 4.55	**0.008** ^**a**^	*ref.*	
≥ 65 years old, *n* = 16	9.50 ± 3.71		−3.362	**0.008**
Ethnicity				
Chinese, *n* = 19	10.11 ± 4.31	0.054^b^	− 2.763	0.030#
Malay, *n* = 38	12.87 ± 4.02		*ref.*	
Indian, *n* = 21	13.33 ± 5.25		0.465	0.701
Others, *n* = 3	9.00 ± 4.36		− 3.868	0.151
Viral Load (log_10_ RNA copies/μl), *n* = 78	*r* = −0.118	0.303^c^	− 0.206	0.584

*TSSS:* Total symptom severity score, *n:* number of patients, *SD:* standard deviation, *ref*. category with the highest number of patients is chosen as reference

^a^*P*-value calculated by Independent Samples t-Test

^b^*P*-value calculated by One-way ANOVA

^c^*P*-value calculated by bivariate correlations

p*: p-value calculated by simple linear regression

*r*: Pearson’s correlation coefficient

*β*: linear regression coefficient

statistically significant comparisons (*p* < 0.05) are in bold

#*p*-value for significance was adjusted by Bonferroni correction to *p* < 0.0083 (0.05/6)

In terms of age, mean TSSS was significantly higher in the young and middle-age adults who were < 65 years old (12.86 ± 4.55) as compared to the elderly population who were ≥ 65 years old (9.50 ± 3.71) (*p* = 0.008) (Table 2). Moreover, when age was modeled as a continuous variable, it had a significant negative correlation with TSSS (*r* = − 0.335, *p* = 0.002). In a simple linear regression model, for the increase of every unit (years) in age, there was a significant decrease of 0.080 unit (score) of TSSS. Thus, our results indicated that age was the only significant predictor of TSSS, with the elderly patients experiencing less severe symptoms than the young and middle-age adults in an outpatient setting. Though, there was no significant difference observed in the estimated enrollment period after symptom onset between patients who were < 65 years old (4.58 ± 3.36 days) and ≥ 65 years old (4.81 ± 2.20 days) (*p* = 0.797).

### The impact of viral load on symptom severity

The performance of the improved one-step RT-qPCR assay targeting the *M2* gene of HMPV was first evaluated using a 6-log_10_ dilution series of HMPV *M2* synthetic oligonucleotide standard. The assay produced a typical amplification plot and a standard curve with a correlation coefficient of 0.999 and amplification efficiency of 96.78%, with a coefficient of determination (*R*^*2*^) of 0.996 (Additional file 3). The intra- and inter-assay variability was estimated within standard range (Additional file 4). Nasopharyngeal viral load in 78/81 HMPV-positive specimens were quantified, with a success rate of 96.5% (Additional file 2). Besides being able to detect all different genotypes and sub-lineages of HMPV, the lowest quantifiable concentration of the assay was estimated at 13 RNA copies/μl, while the highest viral load recorded in our specimen was 731,917 RNA copies/μl.

**Fig. 2:**
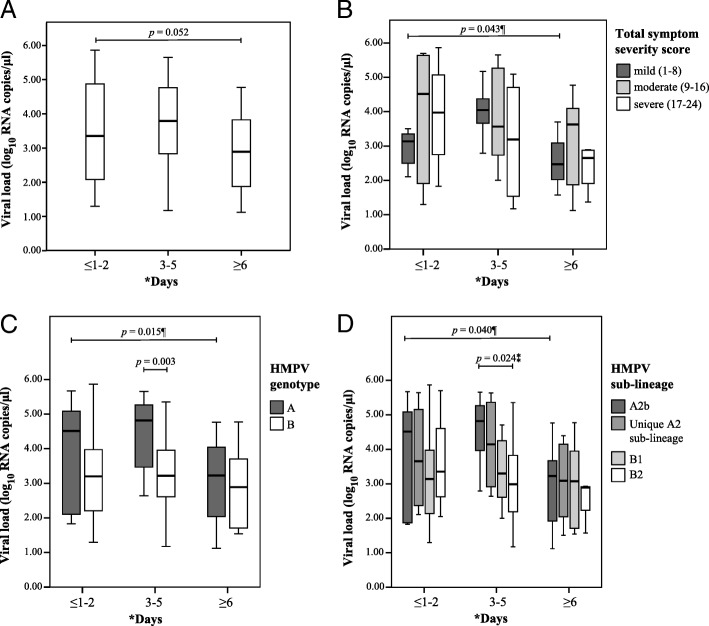
Viral load at different periods of enrollment after symptom onset. **a**) total HMPV-infected patients, **b**) patients with different symptom severity, **c**) patients infected with different HMPV genotypes and **d**) patients infected with different HMPV sub-lineages

At different periods of enrollment after symptom onset (≤1–2 days, 3–5 days, and ≥ 6 days), we observed that patients who enrolled in 3–5 days had the highest viral load (3.77 ± 1.20 log_10_ RNA copies/μl, *n* = 35) compared to those who enrolled in ≤1–2 days (3.55 ± 1.53 log_10_ RNA copies/μl, *n* = 20) and in ≥6 days (2.93 ± 1.16 log_10_ RNA copies/μl, *n* = 23) after symptom onset (Fig. 2a, Additional file 5). Even though no bivariate association between viral load and periods of enrollment after symptom onset was observed (*p* = 0.052), a post-hoc test using the Bonferroni procedure showed that those who enrolled in 3–5 days had higher viral load (*p* = 0.048, insignificant when Bonferroni-corrected *p* < 0.0083 instead of *p* < 0.05 was used) compared to those who enrolled in ≥6 days after symptom onset.

However, the impact of viral load on symptom severity was investigated by performing bivariate correlation between the estimated viral load and TSSS, in which no significant correlation was observed (*r* = − 0.118, *p* = 0.303) (Table 2). Taking into account the different periods of enrollment after symptom onset, there was also no significant correlation observed between viral load and TSSS in these periods (Additional file 5). When the patients’ TSSS was grouped into three categories with scores that range from 1 to 8, 9–16, and 17–24 to reflect the relative symptom severity of mild, moderate, and severe, respectively [20] (Fig. 2b), no significant differences in viral load between the three categories of symptom severity were observed. Though, among the group of patients with a TSSS of 1–8 (indicative of having milder symptoms) (*n* = 19), those who enrolled ≥6 days after symptom onset had a significantly lower viral load (2.58 ± 1.07 log_10_ RNA copies/μl, *n* = 3) compared to those who enrolled 3–5 days after symptom onset (3.99 ± 0.81 log_10_ RNA copies/μl, *n* = 9) (*p* = 0.043), but the difference was insignificant after Bonferroni correction (Fig. 2b, Additional file 5). Overall, our analyses showed that nasopharyngeal viral load could not predict the severity of symptoms caused by HMPV infection, as well as the TSSS scoring system could not predict the amount of viral load in the patient, hence both variables were found to be not correlated in this study.

### Association of viral load with HMPV genetic diversity and demographic factors

By comparing the genetic diversity of HMPV, we observed that after 3–5 days of symptom onset, genotype A patients had a significantly higher viral load (4.44 ± 1.07 log_10_ RNA copies/μl, *n* = 15) compared to genotype B patients (3.27 ± 1.07 log_10_ RNA copies/μl, *n* = 20) (*p* = 0.003) (Fig. 2c, Additional file 5). However, viral load differences between sub-lineages were not significant after Bonferroni correction (*p* = 0.024), though the A2b sub-lineage had a higher viral load (4.55 ± 0.96 log_10_ RNA copies/μl, *n* = 11) compared to B2 (3.07 ± 1.33 log_10_ RNA copies/μl, *n* = 8, *p* = 0.037) sub-lineage during this 3–5 days period (Fig. 2d, Additional file 5). On the other hand, we also observed that genotype A patients who enrolled ≥6 days had a significantly lower viral load (after Bonferroni correction) compared to those who enrolled 3–5 days after symptom onset (*p* = 0.012, post-hoc test with Bonferroni procedure) (Fig. 2c). Lastly, no significant viral load differences were found between the different demographics (sex, ethnicity, and age) of patients at any of the three periods of enrollment after symptom onset (Additional file 5).

## Discussion

Studies assessing the risk factors such as genetic diversity and viral load that contribute to the burden of illness caused by HMPV infection have generally focused on high-risk populations such as hospitalized children [27] or adult patients [28], immunocompromised elderly [29], or hematopoietic cell transplant recipients [30], with limited reports from outpatients presenting with acute respiratory tract symptoms. While individuals seeking and receiving outpatient care may not experience severe respiratory complications, their illnesses contribute significantly to the overall disease burden and transmission of the virus to susceptible individuals. The present study investigated the severity of acute respiratory symptoms caused by HMPV infection in a generally adult outpatient population, and assessed the virological and demographical factors that may be associated with the degree of symptom severity.

A standardized four-category scoring of the severity of common cold symptoms has been used as a complementary measure for the impact of respiratory illness in outpatients [16–19]. By summing up the scores of all individual symptoms [20], we could not observe any significant symptom severity differences (or TSSS) between HMPV genotypes/sub-lineages in the adult outpatients (Table 2), suggesting that the genetic diversity of HMPV as shown in this study played limited role as a predictor for the severity of symptoms in the outpatient settings. Moreover, patients infected with a recently described sub-lineage A2, which was identified based on the estimated genetic distances between sub-lineages in the *F* and *G* genes and was also found in other countries such as China, Vietnam and Cambodia [15], did not show more severe symptoms compared to other sub-lineages. Several previous studies have reported the association of HMPV genetic diversity with disease severity in children. In one report, children infected by genotype A were found to experience more severe acute respiratory illness (based on a scoring method that measures the need for hospitalization, oxygen saturation < 90% at hospital admission, and intensive care unit stay) as compared with genotype B infection [7]. In contrast, a separate study on young children suggested that genotype B infection may result in greater hospitalization rate and higher clinical score (using two scoring methods [7, 31] compared to genotype A [6]. Yet, no significant differences in the severity of illness or clinical manifestation have been reported between HMPV genotypes in children in several other studies [8, 9, 32, 33], which used other scoring methods. The contrasting findings in these studies highlighted that the relationship between HMPV genetic diversity and disease severity remains undetectable, probably due in part to the lack of a consensus and standardized severity scoring method for outpatients or hospitalized patients. Inclusion of other objective assessment including body temperature, chest X-ray and also respiration rate may help to improve the analysis and reduce discordant findings between studies. In addition, the inconclusive association between HMPV genetic diversity and symptom severity could also be due to the low number of HMPV-positive samples in this study as well as several other studies [7, 9], which may reduce the statistical power for associations [34].

It has been described in several studies that HMPV reinfection can occur during an adult’s life due to transient immunity or incomplete protective immune responses for the newly evolved genotype [29, 35], indicating that the elderly individuals are equally susceptible to reinfection. Furthermore, the possible immune dysregulation which lead to a decreased viral clearance in the elderly will further increase their risk of severe symptomatic infection [28] and lead to higher rates of hospitalization [36, 37]. However, unlike previous studies, we found that elderly patients who were ≥ 65 years old had less severe symptoms compared to young and adult patients who were < 65 years old. As our study was conducted in the outpatient clinic, it is possible that elderly patients with severe respiratory symptoms may have opted to seek immediate treatment at inpatient settings, leading to sampling bias. Hence, such contrasting observation may be due to the limited sampling on elderly patients who experience more severe symptoms compared to the young and adult patients. Similarly, even though the Indian ethnic group may appear to be experiencing more severe symptoms (based on higher TSSS score) compared to other ethnic groups in this study (Table 2), such observation needs to be interpreted with caution given the limited number of patients in our cohort. Overall, it remains unclear if host demographic factors could be contributing to a more severe symptom outcome in HMPV infection.

The availability of an up-to-date RT-qPCR assay is important for sensitive, specific and rapid detection and quantification of HMPV. Previously, assays for the detection and quantification of HMPV were developed based on limited genome sequences that belonged to the five known genetic lineages/sub-lineages of HMPV: A1, A2 (A2a and A2b sub-lineage), B1 and B2 [37, 38]. Besides, most published assays were designed to target the conserved regions of the nucleoprotein (*N*) gene [39–41] as it is the highly transcribed and conserved gene [24, 42], even though nucleotide mismatches between primer/probe sets with reference sequences have been reported [28, 41, 43, 44]. Thus, in this study, using an updated alignment of complete reference genomes (*n* = 135), primers/probes were designed to target the conserved region of the *M2* gene with a minimum coverage of 99.3% of the global HMPV sequences (Additional file 1). The viral load in all but three HMPV-positive specimens was successfully captured with the lowest quantifiable concentration estimated at approximately 13 RNA copies/μl, which was more sensitive than previously published methods [38, 43].

High nasopharyngeal HMPV viral load has been implicated as an important risk factor for severe symptoms in children who were hospitalized [12, 45] or admitted for emergency care [46]. However, in this study, no correlation was observed between viral load and symptom severity in adult outpatients (Table 2, Fig. 2 and Additional file 5). Interestingly, it was found that patients infected with genotype A had a significantly higher peak viral load compared to genotype B-infected patients around 3–5 days of symptom onset, suggesting that genotype A may have a better replication fitness (or replication capacity) and higher transmissibility than genotype B during this period. However, the viral load of genotype A-infected patients who enrolled ≥6 days were observed to be significantly lower compared those who enrolled 3–5 days after symptom onset, due to the fact they enrolled much later during the course of infection, in which most viruses would have been cleared by immunity. Interestingly, our findings corroborates with a previous report that demonstrated the differences in replication fitness between HMPV genotypes in vitro and in vivo [47]. The study by Aerts et al. showed that HMPV genotype A replicates to a significantly higher titers than genotype B in LLC-MK2 cells and in the lungs of BALB/c mice on day 4 post-infection, but the viral titers of genotype A decreased more rapidly than genotype B after day 4 [47]. As observed in other viral genotypes/serotypes [48, 49], the differences in replication capacity may contribute to the competitive, transmission and epidemiological fitness differences between HMPV genotypes [50], which in turn may dictate the spread and evolution of both genotypes in the human population.

## Conclusions

This study investigated the impact of HMPV genetic diversity and viral load (estimated using an improved quantification assay) on symptom severity in an adult outpatient cohort presenting with acute respiratory tract symptoms, in which both factors were found not to be associated with a more severe symptom outcome. Significant difference in the viral load dynamics between HMPV genotypes A and B was observed during the course of infection due probably to the difference in viral fitness, which may have important implications on virus transmission.

## Additional files

Additional file 1: Coverage of newly designed primers set and probe in 135 HMPV genomes. (PDF 229 kb)

Additional file 2: Virological, demographical, and clinical information of patients infected with HMPV. (PDF 2165 kb)

Additional file 3: A) Amplification plot of one-step RT-qPCR from this study, showing standard concentration of HMPV oligonucleotide of 2.0 × 10^6^ genomic copies/μl to 2.0 × 10^1^ genomic copies/μl. B) Standard curve showing the amplification efficiency of the assay. (PDF 979 kb)

Additional file 4: Intra and inter variability of the improved RT-qPCR assay for HMPV quantification. (PDF 226 kb)

Additional file 5: Viral load of HMPV-infected patients at different periods of enrollment after symptom onset (PDF 954 kb)

## Abbreviations

Cq: Quantification cycle
CV: Coefficient of variance
FAM: 6-fluorescein amidite
GTR: General time-reversible
HMPV: Human metapneumovirus
MGB: Minor groove binder
ML: Maximum likelihood
NFQ: Non-fluorescent quencher
RT-qPCR: Reverse transcriptase-quantitative polymerase chain reaction
SD: Standard deviation
TSSS: Total symptom severity score
URTI: Upper respiratory tract infection
