# Supplementary material for: The role of human Metapneumovirus genetic diversity and nasopharyngeal viral load on symptom severity in adults
Source: Virol J. 2018 May 23;15:91. doi: 10.1186/s12985-018-1005-8 (PMC5966857; doi:10.1186/s12985-018-1005-8)
Supplement: Supplementary file 1 — Coverage of newly designed primers set and probe in 135 HMPV genomes. (PDF 229 kb) [file 12985_2018_1005_MOESM1_ESM.pdf]

Additional file 1. Coverage of newly designed primers set and probe in 135 HMPV genomes.

Primer/ Probe	Name*	Sequence 5′ to 3′	Genetic region	%Coverage (n = 135)	
				100% identity	One mismatch
Forward	4730f	CGYAARGCTCCRTGCAAATATGAAG	*M2*	99.3	99.3
Reverse	4919r	CTTCTCTRCCTGCDCCTGATATTATTG	*M2*	100.0	100.0
Probe	4796fp	FAM-CACAAYTACTGGAGYTGGC-MGB	*M2*	99.3	99.3

* Nucleotide location was determined based on NC_004148.2.
