# Supplementary material for: The role of human Metapneumovirus genetic diversity and nasopharyngeal viral load on symptom severity in adults
Source: Virol J. 2018 May 23;15:91. doi: 10.1186/s12985-018-1005-8 (PMC5966857; doi:10.1186/s12985-018-1005-8)
Supplement: Supplementary file 2 — Virological, demographical, and clinical information of patients infected with HMPV. (PDF 2165 kb) [file 12985_2018_1005_MOESM2_ESM.pdf]

Additional file 2. Virological, demographical, and clinical information of patients infected with HMPV.

No.	Enrollment Date	ID	HMPV*		Demographics			Days*	Viral Load*		Symptoms								TSSS
			Genotype	Sub-lineage	Sex	Ethnicity	Age		RNA copies/µl	$\log_{10}$ RNA copies/µl	Sneezing	Nasal discharge	Nasal congestion	Cough	Sore throat	Hoarseness of voice	Muscle ache	Headache	
1	27/02/2012	MY/U27/2012	A	Unique A2	Female	Chinese	64	1	127.6181	2.11	None	Mild	None	Severe	None	Mild	None	Mild	6
2	07/03/2012	MY/U78/2012	B	B1	Male	Malay	26	1	4688.0552	3.67	Moderate	Moderate	Severe	Severe	Moderate	Moderate	Moderate	Severe	19
3	09/03/2012	MY/U80/2012	B	B1	Female	Chinese	33	1	81.3143	1.91	None	Severe	Moderate	Severe	None	Moderate	Severe	None	13
4	09/03/2012	MY/U88/2012	B	B2	Female	Chinese	78	1	3190.3176	3.50	None	None	Mild	Severe	None	Mild	Severe	None	8
5	16/03/2012	MY/U134/2012	B	B1	Female	Malay	27	1	19.7194	1.29	Severe	Severe	Moderate	Severe	Severe	Moderate	None	None	16
6	21/03/2012	MY/U167/2012	B	B1	Male	Malay	54	3	2199.3967	3.34	Mild	None	Mild	Severe	Moderate	Mild	Severe	Mild	12
7	23/03/2012	MY/U178/2012	B	B1	Female	Malay	25	2	18903.066	4.28	Moderate	Severe	Severe	Severe	Moderate	Moderate	None	Moderate	17
8	28/03/2012	MY/U203/2012	A	Unique A2	Female	Indian	54	7	14050.9	4.15	Mild	None	None	Mild	Severe	Moderate	Severe	Severe	13
9	28/03/2012	MY/U207/2012	B	B1	Male	Malay	24	4	406.5179	2.61	Mild	Mild	Mild	Moderate	Mild	Moderate	Mild	Moderate	11
10	13/04/2012	MY/U288/2012	B	B1	Female	Malay	63	3	29285.822	4.47	Mild	Mild	None	Severe	None	Mild	Moderate	Mild	9
11	16/04/2012	MY/U306/2012	B	B1	Female	Indian	30	7	276.2403	2.44	Severe	Severe	Severe	Severe	Severe	Severe	Severe	Moderate	23
12	16/04/2012	MY/U319/2012	A	Unique A2	Female	Malay	32	14	32.2699	1.51	Moderate	Moderate	Moderate	Moderate	Severe	Moderate	None	Mild	14
13	07/05/2012	MY/U449/2012	B	B1	Female	Indian	59	3	409.013	2.61	None	None	None	Severe	Severe	Severe	Severe	Severe	15
14	30/05/2012	MY/U576/2012	A	A2b	Female	Chinese	69	2	73.7146	1.87	Mild	None	Mild	Severe	Severe	Mild	None	None	9
15	11/06/2012	MY/U663/2012	B	B2	Male	Chinese	86	7	37.2459	1.57	None	Mild	None	Moderate	None	Mild	None	None	4
16	27/06/2012	MYU752/2012	A	Unique A2	Female	Chinese	19	2	433.3848	2.64	None	None	None	Severe	None	Mild	None	None	4
17	22/08/2012	MY/U1021/2012	B	B1	Male	Indian	80	8	5052.9121	3.70	Mild	None	Moderate	Moderate	None	Mild	Mild	None	7
18	05/09/2012	MY/U1109/2012	B	B1	Male	Indian	31	5	1539.801	3.19	None	Mild	Severe	Severe	Severe	Severe	Severe	Severe	19
19	07/09/2012	MY/U1122/2012	A	A2b	Female	Indian	23	7	4702.193	3.67	None	Severe	Moderate	Severe	Mild	Mild	None	None	10
20	24/09/2012	MY/U1191/2012	A	A2b	Female	Indian	19	4	161590.953	5.21	Mild	None	None	Severe	Severe	Moderate	Severe	Severe	15
21	28/09/2012	MY/U1219/2012	B	B1	Female	Malay	28	3	293.412	2.47	None	None	Severe	Mild	Severe	Moderate	Moderate	Severe	14
22	04/10/2012	MY/U1239/2012	B	B1	Female	Malay	33	3	6239.857	3.80	Moderate	None	Severe	Severe	Mild	Moderate	Mild	Moderate	14
23	15/10/2012	MY/U1315/2012	B	B2	Female	Chinese	36	5	225972.859	5.35	Severe	Severe	Severe	Mild	Moderate	Moderate	None	None	14
24	12/11/2012	MY/U1455/2012	A	Unique A2	Female	Indian	46	3	123368.672	5.09	Mild	Moderate	Moderate	Severe	Severe	Severe	Severe	Severe	20
25	14/11/2012	MY/U1468/2012	A	A2b	Female	Others	69	3	148211.422	5.17	Mild	Mild	None	Moderate	None	Mild	Mild	Mild	7
26	14/11/2012	MY/U1470/2012	A	Unique A2	Female	Malay	34	3	427307.594	5.63	Moderate	Moderate	Moderate	Severe	Severe	Moderate	None	Moderate	16
27	26/11/2012	MY/U1560/2012	A	Unique A2	Male	Others	22	5	433.997	2.64	None	Moderate	Moderate	Moderate	Moderate	Moderate	Moderate	Moderate	14
28	30/11/2012	MY/U1589/2012	A	A2b	Female	Chinese	32	4	18232.566	4.26	None	None	None	Mild	Severe	Mild	None	None	5
29	03/12/2012	MY/U1601/2012	B	B1	Female	Indian	44	4	11146.397	4.05	Mild	Mild	None	Moderate	Mild	None	None	None	5
30	10/12/2012	MY/U1634/2012	A	A2b	Female	Chinese	73	5	327035.406	5.51	Mild	None	None	Severe	Moderate	Moderate	Moderate	Mild	11
31	17/12/2012	MY/U1662/2012	A	A2b	Female	Malay	55	3	4615.89	3.66	Mild	Moderate	None	Moderate	Mild	Moderate	None	None	8
32	24/12/2012	MY/U1692/2012	A	A2b	Female	Malay	35	7	732.626	2.86	Mild	Severe	Moderate	Severe	Severe	Moderate	Severe	None	17
33	24/12/2012	MY/U1698/2012	A	A2b	Female	Malay	62	7	Not detected		None	None	None	Severe	None	Mild	Mild	Mild	6
34	31/12/2012	MY/U1743/2012	A	A2b	Male	Malay	55	7	296.544	2.47	Mild	Moderate	None	Moderate	None	Mild	None	Mild	7
35	07/1/2013	MY/U1784/2013	B	B2	Male	Others	23	2	1582.9263	3.20	Mild	Mild	None	Mild	None	None	Mild	Moderate	6
36	04/2/2013	MY/U1928/2013	A	A2b	Female	Malay	32	2	67.4442	1.83	Moderate	Moderate	Moderate	Severe	Severe	Severe	Mild	Severe	19
37	22/02/2013	MY/U2016/2013	A	A2b	Male	Malay	22	3	23689.369	4.37	Mild	Mild	Mild	Mild	Mild	Mild	Mild	Mild	8
38	27/02/2013	MY/U2054/2013	A	A2b	Female	Chinese	74	7	13.154	1.12	None	Moderate	Moderate	Moderate	None	None	Severe	Mild	10
39	06/03/2013	MY/U2081/2013	A	A2b	Female	Chinese	53	7	4655.516	3.67	Mild	Moderate	Severe	Severe	Mild	Moderate	None	None	12
40	11/03/2013	MY/U2125/2013	B	B1	Male	Malay	59	4	100.447	2.00	Mild	Moderate	Moderate	Severe	Severe	Moderate	Moderate	Mild	16
41	15/03/2013	MY/U2135/2013	A	A2b	Female	Malay	52	4	613.325	2.79	Mild	Mild	Mild	Moderate	None	Mild	Moderate	None	8
42	15/03/2013	MY/U2139/2013	B	B1	Female	Malay	29	7	51.131	1.71	Mild	Moderate	Severe	Severe	Moderate	None	Mild	Severe	15
43	18/03/2013	MY/U2151/2013	A	Unique A2	Female	Malay	55	7	134.615	2.13	Severe	Severe	None	Severe	Mild	Moderate	Mild	Mild	14
44	20/03/2013	MY/U2177/2013	A	Unique A2	Male	Chinese	70	7	24742.322	4.39	Mild	Moderate	Severe	Moderate	Moderate	Moderate	Moderate	Moderate	16
45	12/04/2013	MY/U2281/2013	A	A2b	Female	Malay	42	7	3878.815	3.59	None	Mild	None	Severe	Moderate	Severe	Mild	Moderate	12
46	12/04/2013	MY/U2288/2013	A	A2b	Female	Malay	28	3	65742.992	4.82	Mild	Moderate	Mild	Moderate	Mild	Mild	None	None	8
47	17/04/2013	MY/U2311/2013	A	Unique A2	Female	Indian	70	7	Not detected		Mild	Mild	Mild	Severe	None	None	Mild	None	7
48	22/04/2013	MY/U2329/2013	B	B1	Male	Malay	33	14	34.766	1.54	None	None	Moderate	None	Severe	Moderate	Moderate	Severe	12
49	24/04/2013	MY/U2333/2013	B	B2	Female	Indian	20	7	854.278	2.93	Mild	Mild	None	Severe	Severe	Moderate	Moderate	Mild	13
50	06/05/2013	MY/U2372/2013	A	A2b	Female	Malay	26	2	32612.559	4.51	Mild	Moderate	Moderate	Moderate	Mild	Mild	Moderate	Mild	12
51	05/06/2013	MY/U2472/2013	B	B1	Female	Indian	68	2	1369.047	3.14	None	None	Mild	Mild	Moderate	Mild	None	None	5
52	17/06/2013	MY/U2514/2013	B	B1	Female	Chinese	62	1	229.908	2.36	Mild	Mild	Mild	Severe	None	Moderate	None	None	8
53	05/08/2013	MY/U2658/2013	B	B2	Female	Chinese	74	5	681.7276	2.83	Mild	Mild	Mild	Severe	None	Moderate	Severe	Mild	12
54	09/09/2013	MY/U2776/2013	A	A2b	Male	Malay	47	2	121305.172	5.08	Moderate	Moderate	Moderate	Severe	Mild	Mild	Moderate	None	13
55	11/09/2013	MY/U2780/2013	A	Unique A2	Female	Chinese	71	7	11052.001	4.04	None	Severe	None	Severe	None	Moderate	None	Mild	9

56	23/09/2013	MY/U2830/2013	A	A2b	Male	Chinese	30	4	1866.268	3.27	Moderate	Moderate	None	Severe	Severe	Moderate	Severe	Mild	16
57	25/10/2013	MY/U2953/2013	B	B1	Male	Indian	25	7	8847.6885	3.95	Severe	Severe	Severe	Severe	Mild	Moderate	None	None	15
58	01/11/2013	MY/U2988/2013	B	B2	Male	Indian	75	4	7222.648	3.86	None	None	None	Severe	Mild	None	Severe	None	7
59	04/11/2013	MY/U3002/2013	B	B2	Female	Malay	56	3	14.986	1.18	Severe	Severe	Mild	Moderate	Moderate	Moderate	Moderate	Moderate	17
60	08/11/2013	MY/U3021/2013	A	Unique A2	Male	Chinese	34	2	46243.234	4.67	None	None	None	Mild	None	None	Mild	Severe	5
61	13/11/2013	MY/U3051/2013	B	B2	Female	Malay	63	4	683.146	2.83	Mild	None	None	Mild	None	Mild	None	Mild	4
62	15/11/2013	MY/U3059/2013	A	Unique A2	Male	Indian	51	2	436264.781	5.64	Mild	Mild	Moderate	Severe	None	Moderate	None	Mild	10
63	25/11/2013	MY/U3123/2013	B	B1	Male	Malay	64	7	59303.898	4.77	Mild	Moderate	Mild	Severe	Severe	Moderate	None	Mild	13
64	27/11/2013	MY/U3126/2013	B	B1	Female	Malay	51	4	1771.071	3.25	Mild	None	Moderate	Severe	Severe	Severe	Mild	Severe	16
65	27/11/2013	MY/U3142/2013	B	B2	Female	Malay	22	3	34.675	1.54	Severe	Severe	Severe	Severe	Mild	Moderate	Moderate	Moderate	19
66	11/12/2013	MY/U3206/2013	B	B1	Female	Indian	75	4	50904.121	4.71	Moderate	Severe	Moderate	Severe	Mild	Moderate	Severe	Mild	17
67	23/12/2013	MY/U3280/2013	B	B2	Female	Malay	22	5	6155.402	3.79	None	Mild	Moderate	Mild	Moderate	Mild	Moderate	Severe	12
68	10/01/2014	MY/U3350/2014	B	B2	Female	Indian	26	4	1375.908	3.14	Mild	Mild	Mild	Severe	Moderate	None	Severe	Severe	14
69	15/01/2014	MY/U3373/2014	A	A2b	Male	Malay	25	7	23.446	1.37	Mild	Severe	Severe	Severe	Severe	Moderate	Severe	Severe	21
70	24/01/2014	MY/U3417/2014	B	B1	Male	Indian	47	2	731916.625	5.86	Moderate	None	Moderate	Severe	Severe	Severe	Severe	Mild	17
71	29/01/2014	MY/U3442/2014	A	A2b	Male	Malay	19	2	469040.906	5.67	Severe	Mild	Moderate	Mild	Mild	Mild	None	None	9
72	07/02/2014	MY/U3466/2014	B	B2	Female	Chinese	21	2	498311.062	5.70	None	Severe	Moderate	Severe	Mild	Mild	None	Mild	11
73	14/02/2014	MY/U3509/2014	B	B2	Female	Malay	66	3	Not detected		None	None	Mild	Severe	Severe	Moderate	Severe	Moderate	14
74	17/03/2014	MY/U3645/2014	B	B2	Male	Indian	56	7	775.8019	2.89	None	Severe	Severe	Severe	Severe	Severe	Severe	Severe	21
75	26/03/2014	MY/U3700/2014	A	Unique A2	Female	Chinese	25	4	1568.691	3.20	Severe	Severe	Severe	Severe	Severe	Severe	Mild	None	19
76	28/03/2014	MY/U3711/2014	A	A2b	Male	Malay	37	3	449996.844	5.65	Mild	Moderate	Moderate	Moderate	Moderate	Moderate	Severe	Mild	15
77	02/04/2014	MY/U3730/2014	A	A2b	Male	Malay	70	5	209055.219	5.32	Mild	Mild	None	Moderate	Mild	Mild	Moderate	Mild	9
78	02/04/2014	MY/U3732/2014	A	A2b	Female	Indian	60	7	58324.957	4.77	Mild	Severe	None	Moderate	None	None	Severe	Severe	12
79	04/04/2014	MY/U3735/2014	A	Unique A2	Male	Indian	55	14	110.123	2.04	None	None	Moderate	Severe	Severe	Mild	Severe	Severe	15
80	14/05/2014	MY/U3893/2014	B	B2	Male	Malay	54	2	111.723	2.05	Moderate	None	None	Severe	None	Moderate	Severe	Moderate	12
81	19/05/2014	MY/U3925/2014	B	B1	Male	Malay	44	5	28361.789	4.45	None	Mild	None	Severe	Mild	Mild	Moderate	Severe	11

HMPV*: Detection of HMPV by xTAG RVP *FAST* multiplex RT-PCR assay and PCR assay on F and G genes for phylogenetic classification (1);
Viral Load*: Viral load quantification by in-house HMPV-specific RT-qPCR assay;
Days*: Estimated number of days elapsed between symptom onset and enrollment date;
TSSS: Total symptom severity score
Unique A2: Unique A2 sub-lineage

Reference

1. Chow WZ, Chan YF, Oong XY, Ng LJ, Nor'E SS, Ng KT, et al. Genetic diversity, seasonality and transmission network of human metapneumovirus: identification of a unique sub-lineage of the fusion and attachment genes. Sci Rep. 2016;6:27730.
