# Supplementary figures and images for: The role of human Metapneumovirus genetic diversity and nasopharyngeal viral load on symptom severity in adults
Source: Virol J. 2018 May 23;15:91. doi: 10.1186/s12985-018-1005-8 (PMC5966857; doi:10.1186/s12985-018-1005-8)

**A**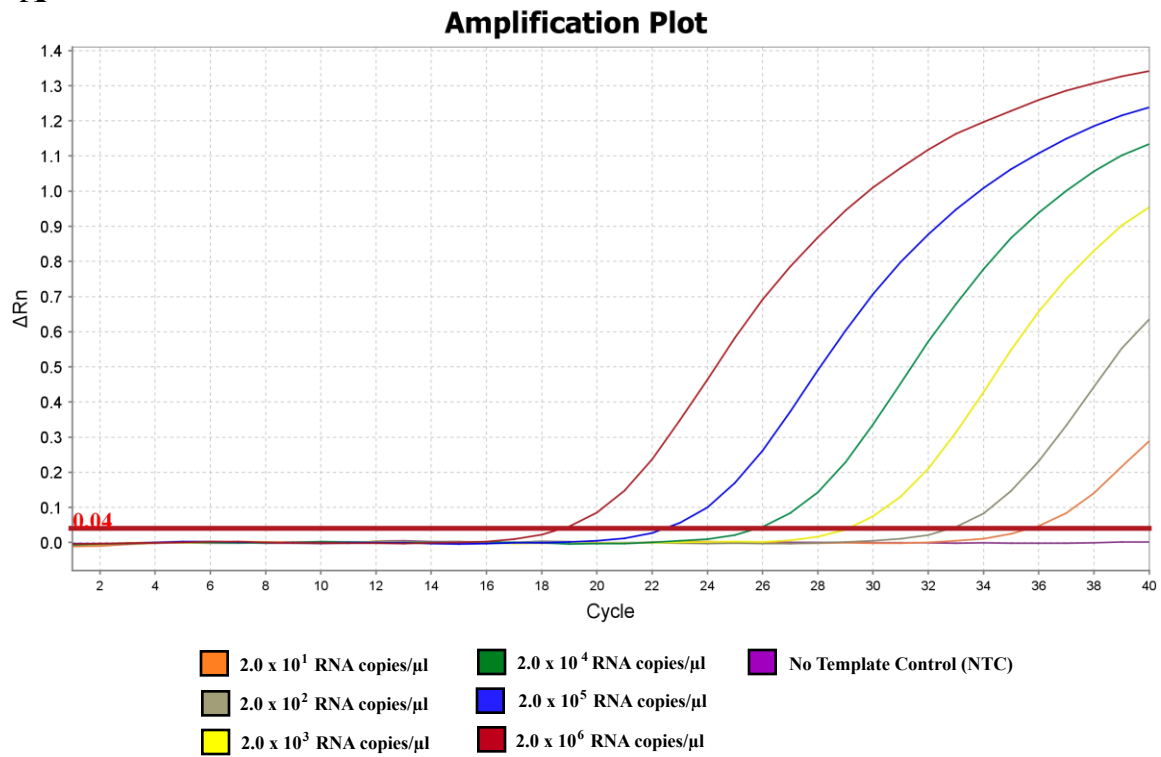**B**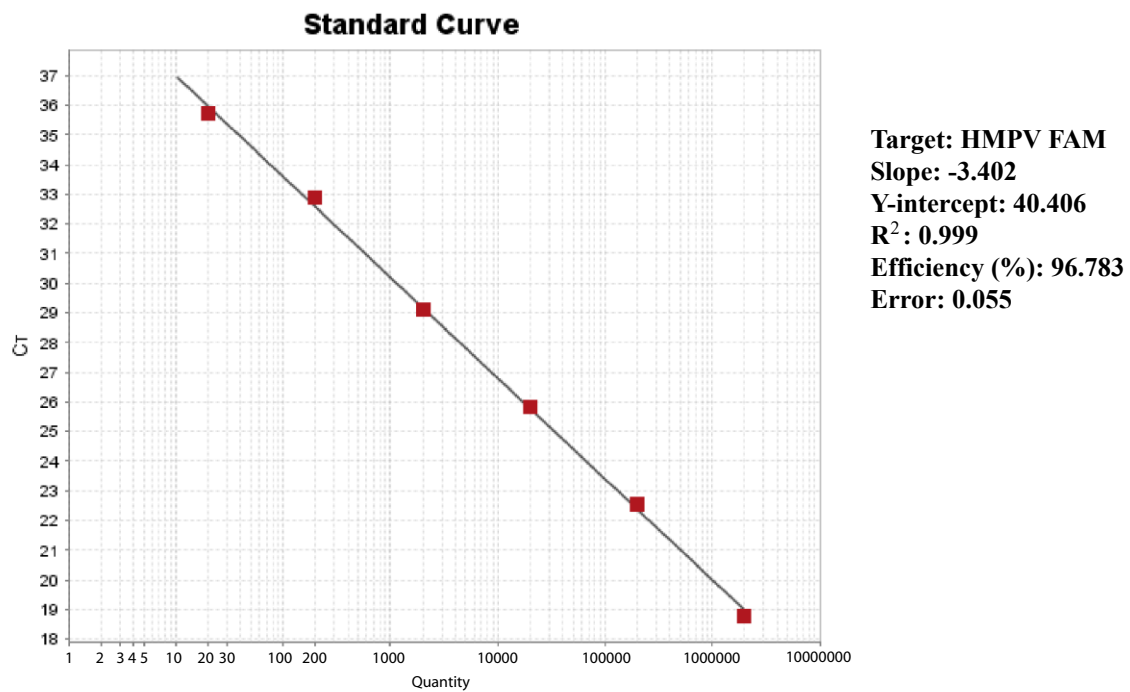

Supplement: Supplementary file 3 — A) Amplification plot of one-step RT-qPCR from this study, showing standard concentration of HMPV oligonucleotide of 2.0 × 10^6^ genomic copies/μl to 2.0 × 101 genomic copies/μl. B) Standard curve showing the amplification efficiency of the assay. (PDF 979 kb) [file 12985_2018_1005_MOESM3_ESM.pdf]
