# Supplementary material for: The role of human Metapneumovirus genetic diversity and nasopharyngeal viral load on symptom severity in adults
Source: Virol J. 2018 May 23;15:91. doi: 10.1186/s12985-018-1005-8 (PMC5966857; doi:10.1186/s12985-018-1005-8)
Supplement: Supplementary file 4 — Intra and inter variability of the improved RT-qPCR assay for HMPV quantification. (PDF 226 kb) [file 12985_2018_1005_MOESM4_ESM.pdf]

Additional file 4. Intra and inter variability of the improved RT-qPCR assay for HMPV quantification.

	Viral load (RNA copies/µl)					
**Assay**	**1**	**2**	**3**	**Mean**	**SD**	**%CV**
**Intra**						
2.0 x 10^3 RNA copies/µl	2,290.023	2,162.963	2,112.960	2188.65	91.28	4.17
2.0 x 10^1 RNA copies/ µl	20.339	12.047	10.420	14.27	5.32	37.28
NTC	Negative	Negative	Negative			
**Inter**						
2.0 x 10^3 RNA copies/µl	2,290.023	2259.549	2435.636	2328.40	94.11	4.04
2.0 x 10^1 RNA copies/µl	20.339	26.870	10.090	19.10	8.46	44.29

NTC: no template control; SD: standard deviation; CV: coefficient of variance
