# Supplementary material for: The role of human Metapneumovirus genetic diversity and nasopharyngeal viral load on symptom severity in adults
Source: Virol J. 2018 May 23;15:91. doi: 10.1186/s12985-018-1005-8 (PMC5966857; doi:10.1186/s12985-018-1005-8)
Supplement: Supplementary file 5 — Viral load of HMPV-infected patients at different periods of enrollment after symptom onset (PDF 954 kb) [file 12985_2018_1005_MOESM5_ESM.pdf]

Additional file 5. Viral load of HMPV-infected patients at different periods of enrollment after symptom onset.

					**Estimated no. of days elapsed between symptom onset and enrollment date**												
	**VL, n=78**				**≤1-2 Days, n=20**				**3-5 Days, n=35**				**≥ 6 Days, n=23**				
**Characteristics**	**mean ± SD**	***p****	***β***	***p*****	**VL, mean ± SD**	***p****	***β***	***p*****	**VL, mean ± SD**	***p****	***β***	***p*****	**VL, mean ± SD**	***p****	***β***	***p*****	***p#***
**TSSS**	r = -0.118	0.303[a]	-0.034	0.303	r = 0.038	0.873[a]	0.012	0.873	r = -0.206	0.235[a]	-0.056	0.235	r = -0.092	0.677[a]	-0.023	0.677	
mild (1-8), n=19	3.43 ± 0.99	0.569	-0.149	0.682	3.08 ± 0.85	0.627	-0.660	0.416	3.99 ± 0.81	0.380	0.112	0.818	2.58 ± 1.07	0.476	-0.541	0.472	0.043¶
moderate (9-16), n=45	3.58 ± 1.40		ref.		3.75 ± 1.91		ref.		3.87 ± 1.23		ref.		3.12 ± 1.26		ref.		0.271
severe (17-24), n=14	3.15 ± 1.44		-0.429	0.294	3.91 ± 1.67		0.163	0.865	3.15 ± 1.59		-0.718	0.290	2.39 ± 0.71		-0.732	0.278	0.359
**HMPV genotype**																	
A, n=38	3.75 ± 1.40	0.065	-0.549	0.065	3.78 ± 1.64	0.556	-0.419	0.556	4.44 ± 1.07	**0.003**	-1.167	**0.003**	2.99 ± 1.21	0.769	-0.151	0.769	**0.015¶**
B, n=40	3.20 ± 1.19		ref.		3.36 ± 1.48		ref.		3.27 ± 1.07		ref.		2.83 ± 1.14		ref.		0.583
**HMPV sub-lineage**																	
A2b, n=24	3.86 ± 1.40	0.266	0.593	0.117	3.79 ± 1.82	0.924	0.577	0.557	4.55 ± 0.96	0.024‡	1.138	0.018‡	2.94 ± 1.24	0.915	-0.079	0.907	0.040¶
Unique A2 sub-lineage, n=14	3.56 ± 1.41		0.299	0.495	3.76 ± 1.67		0.545	0.603	4.14 ± 1.45		0.727	0.255	3.04 ± 1.28		0.025	0.972	0.497
B1, n=25	3.26 ± 1.16		ref.		3.22 ± 1.55		ref.		3.41 ± 0.89		ref.		3.02 ± 1.31		ref.		0.804
B2, n=15	3.09 ± 1.28		-0.172	0.689	3.61 ± 1.53		0.396	0.705	3.07 ± 1.33		-0.346	0.491	2.46 ± 0.77		-0.555	0.532	0.534
**Sex**																	
Male, n= 29	3.63 ± 1.34	0.402	-0.261	0.402	4.48 ± 1.38	0.051	-1.553	0.051	3.70 ± 1.15	0.814	0.105	0.814	2.87 ± 1.26	0.846	0.098	0.846	0.079
Female, n=49	3.37 ± 1.29		ref.		2.93 ± 1.33		ref.		3.81 ± 1.25		ref.		2.97 ± 1.13		ref.		0.064
**Ethnicity**																	
Chinese, n= 19	3.37 ± 1.38	0.472	0.080	0.831	3.09 ± 1.42	0.411	-0.455	0.518	4.07 ± 1.16	0.785	0.494	0.402	2.95 ± 1.50	0.226	0.519	0.420	0.331
Malay, n=36	3.29 ± 1.40		ref.		3.55 ± 1.63		ref.		3.58 ± 1.32		ref.		2.44 ± 1.14		ref.		0.111
Indian, n=20	3.85 ± 1.09		0.565	0.129	4.88 ± 1.51		1.332	0.216	3.98 ± 0.96		0.404	0.446	3.39 ± 0.88		0.954	0.089	0.106
Others, n=3	3.67 ± 1.33		0.383	0.631	3.20 ± 0.00		-0.349	0.832	3.90 ± 1.79		0.327	0.726	0.00 ± 0.00		-	-	0.802
**Age**	r = -0.013	0.913[a]	-0.001	0.931	r = -0.238	0.312[a]	-0.019	0.312	r = 0.097	0.579[a]	0.006	0.579	r = 0.192	0.379[a]	0.011	0.379	
<65 years old, n=64	3.43 ± 1.31	0.622	ref.		3.67 ± 1.60	0.395		ref.	3.61 ± 1.19	0.076		ref.	2.91 ± 1.10	0.932		ref.	0.140
≥65 years old, n=14	3.62 ± 1.40		0.193	0.622	2.84 ± 0.86		-0.839	0.395	4.57 ± 1.03		0.959	0.076	2.97 ± 1.51		0.052	0.932	0.079
**Total, n=78**					3.55 ± 1.53				3.77 ± 1.20				2.93 ± 1.16				0.052

VL: viral load (log_{10} RNA copies/µl); TSSS: Total symptom severity score; SD: standard deviation; ref.: category with the highest number of patients is chosen as reference;
p*: p-value calculated by Independent-Samples t-Test/One-way ANOVA to determine significant mean differences between groups;
[a]p-value calculated by bivariate correlations; r: Pearson's correlation coefficient;
*p***: p-value calculated by simple linear regression; *β*: linear regression coefficient
p#: p-value calculated by One-way ANOVA to determine significant mean differences between different periods of enrollment after onset of symptoms;
Statistically significant comparisons ($p < 0.05$) are in bold;
¶: p-value for significance was adjusted by Bonferroni correction to $p < 0.0167$ (0.05/3);
‡: p-value for significance was adjusted by Bonferroni correction to $p < 0.0083$ (0.05/6).
